# The Clinical Impact of Anti-HLA Donor Specific Antibody Detection Through First Year Screening on Stable Kidney Transplant Recipients

**DOI:** 10.3389/ti.2022.10094

**Published:** 2022-03-17

**Authors:** Akhil Sharma, Dana R. Jorgensen, Rajil B. Mehta, Puneet Sood, Chethan M. Puttarajappa, Christine M. Wu, Amit D. Tevar, Michele Molinari, Adriana Zeevi, Sundaram Hariharan

**Affiliations:** ^1^ Department of Medicine, University of Pittsburgh and University of Pittsburgh Medical Center, Pittsburgh, PA, United States; ^2^ Department of Surgery, University of Pittsburgh and University of Pittsburgh Medical Center, Pittsburgh, PA, United States; ^3^ Department of Immunology, University of Pittsburgh and University of Pittsburgh Medical Center, Pittsburgh, PA, United States; ^4^ Department of Pathology, University of Pittsburgh and University of Pittsburgh Medical Center, Pittsburgh, PA, United States

**Keywords:** donor-specific antibodies, screening, kidney transplant, graft survival, rejection

## Abstract

Anti-HLA Donor Specific Antibody (DSA) detection post kidney transplant has been associated with adverse outcomes, though the impact of early DSA screening on stable patients remain unclear. We analyzed impact of DSA detection through screening in 1st year stable patients (*n* = 736) on subsequent estimated glomerular filtration rate (eGFR), death censored graft survival (DCGS), and graft failure (graft loss including return to dialysis or re-transplant, patient death, or eGFR < 20 ml/min at last follow up). Patients were grouped using 1st year screening into DSA+ (Class I, II; *n* = 131) or DSA- (*n* = 605). DSA+ group were more DR mismatched (*p* = 0.02), more sensitized (cPRA ≥90%, *p* = 0.002), less Caucasian (*p* = 0.04), and had less pre-emptive (*p* = 0.04) and more deceased donor transplants (*p* = 0.03). DSA+ patients had similar eGFR (54.8 vs. 53.8 ml/min/1.73 m^2^, *p* = 0.56), DCGS (91% vs. 94%, *p* = 0.30), and graft failure free survival (76% vs. 82%, *p* = 0.11). DSA timing and type did not impact survival. Among those with a protocol biopsy (*n* = 515), DSA detected on 1st year screening was a predictor for graft failure on multivariate analysis (1.91, 95% CI 1.03–3.55, *p* = 0.04). Overall, early DSA detection in stable patients was an independent risk factor for graft failure, though only among those who underwent a protocol biopsy.

## Graphical Abstract

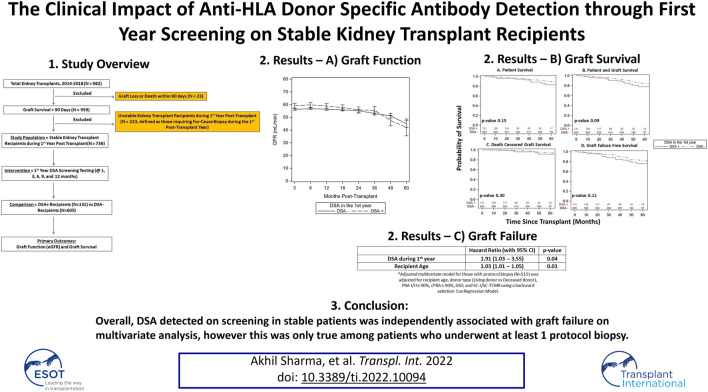

## Introduction

Anti-donor human leukocyte antigen (HLA) specific antibody (DSA) development after kidney transplant is associated with poor clinical outcomes (1–4). Specifically, DSA has been associated with Antibody Mediated Rejection (ABMR) and T-Cell Mediated Rejection (TCMR), with early rejection linked to inferior outcomes (5–11). DSA detection also has been correlated with worse transplant survival, though many with DSA will still have a functioning transplant at 5 years (∼83%) (12). Studies assessing DSA have been varied in population and testing indication, often mixing both for-cause and screening testing. Further, DSA testing is not standardized resulting in variation between laboratories (13–15). These factors have limited assessment of DSA testing as a screening tool in stable patients. With increased efforts to curb health care costs, magnified by an ongoing pandemic, each test ordered and performed must add value to care provided (16–20).

The impact of early DSA screening on patients with stable kidney function without pre-existing DSA at transplant remains unclear and has been identified as a topic requiring study (21–23). To address the impact of early post-transplant DSA screening, we analyzed DSA detected on screening within the 1st year in stable kidney transplant patients and examined correlations with primary outcomes of kidney function and survival. We also analyzed secondary outcomes of subclinical events in the 1st year using protocol biopsies and clinical events beyond the 1st year using for-cause biopsies. We hypothesized that DSA detected on screening in stable 1st year patients would not be associated with inferior survival or function.

## Materials and Methods

### Study Population

**FIGURE 1:**
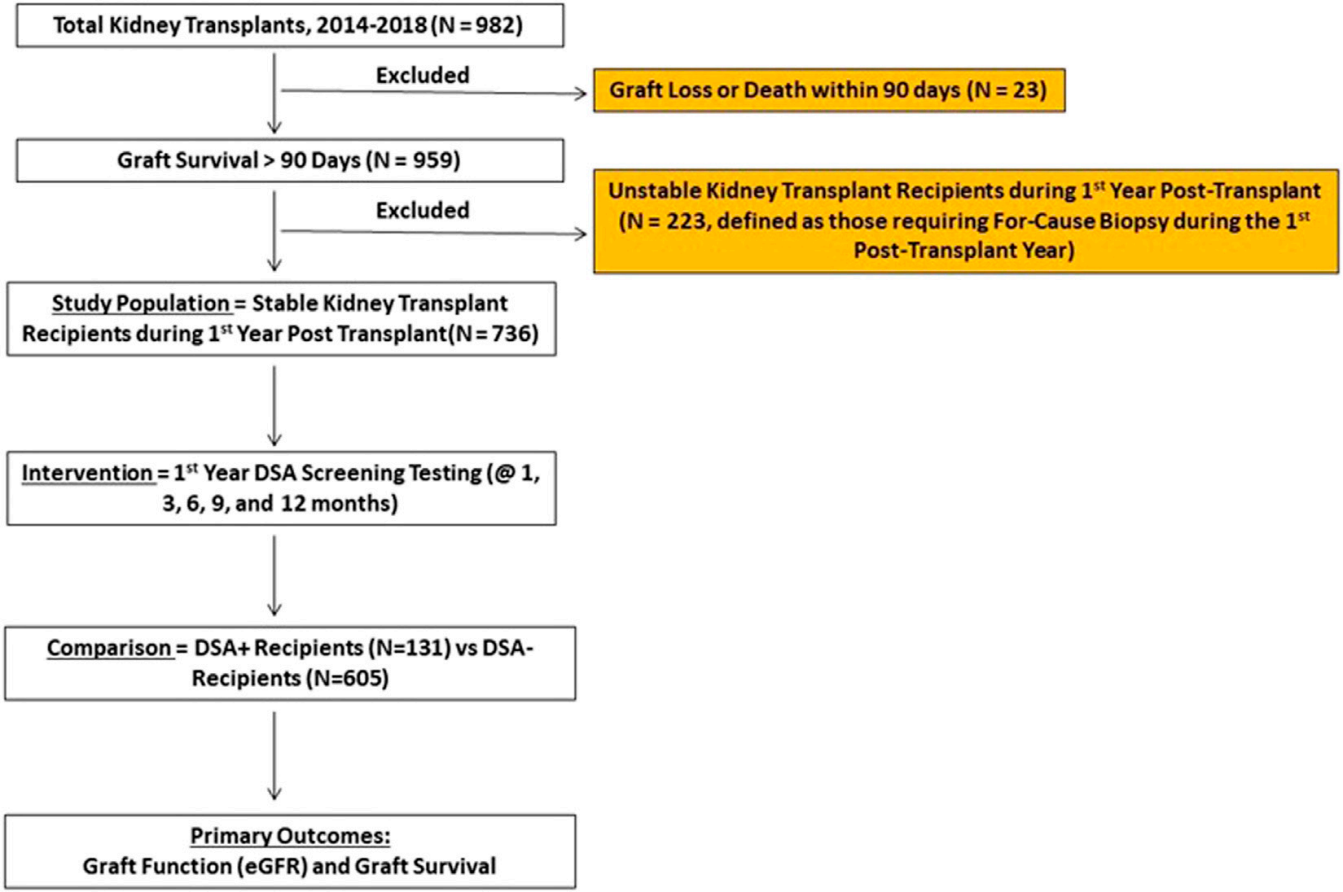
Schematic representation of the study which shows 982 patients who had kidney alone transplant from 2014–2018 and exclusion of 246 patients. The remaining 736 patients with a stable 1st year kidney transplant formed the study cohort and were divided into DSA+ (N = 131, 18%) versus DSA- (N = 605, 82%) based on 1st year surveillance DSA testing. Primary clinical outcomes were assessed as noted.

We studied 982 adult patients who underwent kidney transplant alone (ABO compatible and DSA absent, flow crossmatch (FC) negative at the time of transplant based on last serum within <30 days of transplant) from January 1, 2014 to December 31, 2018 at the Thomas E. Starzl Transplantation Institute—University of Pittsburgh. Repeat kidney and kidney after other solid organ transplant patients were included. We excluded those with early graft loss or death (<90 days, *n* = 23) or an unstable 1st year course (defined as those requiring for-cause biopsy in 1st year, *n* = 223) to limit for-cause DSA testing that often accompanies for-cause biopsies and graft dysfunction. The remaining 736 patients served as our primary study cohort (Figure 1).

### DSA Monitoring

DSA was tested within the 1st year (1, 3, 6, 9, 12 months) per our center’s screening protocol, at time of any biopsy, and annually until 5 years. DSA was considered newly detected as last serum sample available at time of transplant was DSA negative (prior serum was not analyzed). DSA was measured using One Lambda LABScreen™ single antigen bead assay and considered positive if adjusted mean fluorescent intensity (MFI) was ≥1,000 units based on our HLA lab’s designation. A single positive DSA reading (for either class) was considered as a single positive and multiple positive DSA tests for the same class separated in time were considered as multiple positive.

### Immunosuppression

Induction was mainly with thymoglobulin and rarely with basiliximab (if 0% calculated panel reactive antibody [cPRA], 0 antigen mismatch, and a living donor [LD] transplant recipient). For maintenance, majority were on mycophenolate mofetil and calcineurin inhibitor (mainly Tacrolimus) with a minority also on prednisone (those with cPRA ≥ 90% or those on prednisone prior, 5 mg daily or their dose prior to transplant). Prednisone (5 mg daily) was subsequently added to maintenance regimen for any rejection episodes (clinical or subclinical). There was no systematic center protocol for adjusting maintenance immunosuppression based on DSA detection alone.

### Biopsies

Protocol biopsies were recommended to all patients at ∼3 and 12 months post-transplant unless contraindicated. Potential contraindications included those patients on systemic anticoagulation, those on dual anti-platelet therapy, those with intrabdominal kidney location, those who received *en bloc* kidneys, those with active malignancy or serious infection at time of scheduled protocol biopsy, or those lacking transportation. Additionally, as with any medical procedure, patients had the option to decline recommendation to undergo a protocol biopsy after risks and benefits were thoroughly discussed. Biopsies were scored using Banff 2013 and later 2017 classification (24, 25). For-cause biopsies were done for renal dysfunction (rise in serum creatinine >25% from baseline and/or new or worsening proteinuria [>1 g/day and/or >1 g/g urine protein to creatinine ratio]), but not for isolated DSA detection alone.

### Allograft Histology

Protocol biopsy findings were defined as no inflammation (NI, Banff t score 0 + i/ti score 0), subclinical inflammation (SCI, minimal inflammation [MI] Banff t score <0 + i/ti score ≥0 or Banff Borderline Changes [BBC] Banff t score >0 + i/ti score ≥0 and <1A TCMR), and subclinical TCMR (SC-TCMR, ≥1A TCMR). Those with subclinical ABMR (SC-ABMR) were included within these three groups using associated findings (NI, SCI, or SC-TCMR) and were also analyzed separately. Protocol biopsies were also grouped based on timing and maximum grade (highest grade noted on any 1st year protocol biopsy). For-cause biopsies beyond the 1st year were defined as negative (no pathologic findings), inflammation (MI or BBC), rejection (≥1A TCMR and/or ABMR), and non-alloimmune events (urinary tract infection, BK virus nephropathy, acute tubular injury, glomerulonephritis, secondary oxalate nephropathy).

### Follow-Up

The median follow up was ∼3.3 years (Table 1).

**TABLE 1:** Recipient and donor demographics and transplant characteristics of kidney transplant recipients and post-transplant events such as delayed graft function and biopsy rates among study recipients with who had a stable 1st year post-transplant course with DSA+ and DSA-.

	Total (N = 736)	DSA- (N = 605)	DSA+ (N = 131)	*p*-value
Recipient age (years, mean/SD)	52 (14)	53 (14)	51 (12)	0.12
Recipient gender (% male)	59	60	53	0.12
Recipient race (% caucasian)	76	78	68	**0.04**
Body mass index at transplant (kg/m^2^, Mean/SD)	28.7 (5.7)	28.5 (5.7)	29.6 (5.9)	0.05
Preemptive transplant (%)	19	20	12	**0.04**
Cause of end stage kidney disease				
Diabetes mellitus %	23	22	25	0.43
Hypertension %	18	19	15	0.23
Polycystic kidney disease %	11	11	12	0.99
Glomerulonephritis %	5	5	3	0.35
Other/unknown %	43	43	45	0.78
Prior kidney transplant (%)	17	16	21	0.17
Any prior transplant (%)	25	25	24	0.35
Deceased donor %	68	67	76	**0.03**
Donor age (years, mean/SD)	40 (14)	40 (14)	39 (13)	0.66
Donor gender (% male)	55	55	56	0.86
Donor race (% Caucasian)	89	89	86	0.50
Cold ischemia time (minutes, median/IQR)	506 (88–792)	497 (85–792)	544 (204–782)	0.26
KDPI % (Mean/SD)	42 (25)	43 (25)	41 (26)	0.59
% with panel reactive antibody class I ≥ 90%	5	5	7	0.25
% with panel reactive antibody class II ≥ 90%	6	6	6	0.83
% with calculated panel reactive antibody ≥ 90%	15	13	24	**0.002**
Total HLA mismatches (median/IQR)	4 (3–5)	4 (3–5)	4 (4–5)	0.11
DR mismatches (median/IQR)	1 (1–2)	1 (1–2)	1 (1–2)	**0.02**
Cytomegalovirus D+/R- (%)	21	22	16	0.28
Epstein-barr virus D + R- (%)	5	5	7	0.50
Delayed graft function (%)	16	16	17	0.76
At least 1 protocol biopsy (%)	70	69	73	0.36
3 month protocol biopsy (%)	64	63	66	0.46
12 month protocol biopsy (%)	55	54	57	0.61
Biopsy anytime during study (%)	77	76	79	0.41
Any DSA detected beyond 1 year (%)	15	10	39	<0.001
Median follow up (days, median/IQR)	1,199 (808–1,640)	1,204 (805–1,646)	1,146 (832–1,523)	0.44

Bold values considered statistically significant with *p*-value < 0.05.

### Outcome Measures

Primary outcomes were kidney function (estimated glomerular filtration rate [eGFR] using CKD-EPI formula) and survival (patient, combined patient and graft, death censored graft survival [DCGS], and graft failure [defined as graft loss with return to dialysis or re-transplant, death, or eGFR < 20 ml/min at last follow up] free survival). Secondary outcomes were subclinical events (SCI, SC-TCMR, SC-ABMR, mean cumulative acute scores [defined as sum of Banff i/ti, t, g, ptc, and v scores], and mean IFTA score) within 1st year and clinical events (rejection, inflammation, non-alloimmune events) beyond 1st year.

### Ethical Guidelines

Patient information was obtained through specified personnel at Thomas E. Starzl Transplantation Institute as regulated by the institutional review board (IRB) at the University of Pittsburgh. The institution maintains a prospectively collected electronic database of all kidney transplant patients. The studies involving human participants were reviewed and approved by the University of Pittsburgh IRB and the patients were not required to provide written consent for this study per the University of Pittsburgh IRB. We collected data under IRB number PRO-13060220. The activities reported are consistent with the Principles of the Declaration of Istanbul as outlined in the “Declaration of Istanbul on organ trafficking and Transplant Tourism” and Declaration of Helsinki.

### Statistical Methods

Analysis was completed using SAS 9.4 (SAS Institute Inc., Cary NC). Continuous variables are presented as mean ± standard deviation for normally distributed data and median with interquartile range for nonparametric data. Differences in baseline and transplant variables were assessed using analysis of variance and chi-square tests. We evaluated differences in recipient and donor demographics (age, race, gender), and other variables [body mass index (BMI) at transplant, preemptive transplant, End Stage Kidney Disease (ESKD) cause, cold ischemia time (CIT), Kidney Donor Prognostic Index (KDPI), donor type [deceased donor (DD) vs. LD], PRA (panel reactive antibody) Class I/II, cPRA, HLA mismatch (A, B, DR), Cytomegalovirus (CMV)/Epstein Barr Virus (EBV) serostatus, delayed graft function (DGF), biopsy accrual rates] between groups (Table 1). Linear mixed model was used to assess eGFR with a serum creatinine value of 8 mg/dl assigned for graft loss. Covariates for multivariate analysis were identified and evaluated for inclusion before modeling. All multivariate analysis included recipient age, donor type (LD vs. DD), PRA I/II ≥ 90%, cPRA ≥ 90%, DGF, and SCI/SC-TCMR using a backward selection Cox Regression Model with variables with *p* < 0.2 included in the model. Survival (patient, graft, graft failure free) was examined by Kaplan Meier method with survival curves compared by Log rank test. Adjusted Bonferroni *p*-values were used with multiple log-rank comparisons. We examined relationship between DSA and 1st year protocol biopsy findings on eGFR in an exploratory analysis. A *p*-value <0.05 was considered statistically significant.

## Results

### Patient Demographics

The DSA+ cohort included 131 patients (18%) with at least one positive screening DSA test (class I and/or II) during the 1st year and the remaining 605 patients (82%) were the DSA- cohort. DSA+ patients had less Caucasians (68% vs. 78%, *p* = 0.04), fewer were pre-emptive (12% vs. 20%, *p* = 0.04), more were deceased donor (76% vs. 67%, *p* = 0.03), more were anti-HLA sensitized (% cPRA ≥ 90%, 24% vs. 13%, *p* = 0.002), more were DR mismatched (1 [1–2] vs. 1 [1–2], *p* = 0.02), and more had DSA detected (persisting from 1st year or new) beyond 1 year (39% vs. 10%, *p* < 0.001) (Table 1). Other donor and recipient variables as well as protocol biopsy accrual rates were all similar. DSA detection was comparable for those with and without protocol biopsy (19% with vs. 16% without, *p* = 0.36), but those without protocol biopsy were more likely to have diabetic ESKD, prior transplant, increased anti-HLA sensitization, received DD transplant, longer CIT, and less likely to have had a preemptive transplant (Supplementary Table S1).

### DSA Characteristics

DSA were primarily detected ∼1–4 months post-transplant (class I at 40 days [30–108] and class II at 38 days [31–135]) with 77% of first DSA detected within 100 days (Table 2). Additionally, 60% of DSA+ patients had at least one multiple positive DSA for the same class and 18% of DSA+ patients had both class I and II DSA detected within 1st year.

**TABLE 2:** Donor specific antibody (DSA) characteristics for DSA+ patients who underwent kidney transplant and had a stable 1st year post-transplant course.

DSA characteristic	DSA+ (N = 131)
# of Class I tests during 1st year^a^	8 (6–10)
# of Class II tests during 1st year^a^	8 (6–10)
# of + Class I tests during 1st year^a^	1 (0–2)
# of + Class II tests during 1st year^a^	1 (0–3)
Time to + Class I test (days)^a^	41 (30–108)
Time to + Class II test (days)^a^	38 (31–135)
Class I + % during 1st year	57
Class II + % during 1st year	62
DSA detected within 100 days (%)	77
Single positive DSA (%)	46
Multiple positive DSA (%)	60
DSA type	
Negative (%)	0
Class I (%)	38
Class II (%)	44
Class I and Class II (%)	18

a Median and IQR, noted.

### Protocol Biopsy Findings Within 1st Year

Protocol biopsy results for those with at least one protocol biopsy (*n* = 515, 70%) are shown in Table 3. DSA+ patients had similar protocol biopsy rates vs. DSA- patients (73% [96 patients, 159 biopsies] vs. 69% [419 patients, 696 biopsies], *p* = 0.36). Mean cumulative acute and IFTA scores were similar at 3 and 12 months. Frequency of protocol biopsies with NI, SCI, and SC-TCMR were comparable among groups based on 3-months, 12-months, and maximum 1st year grade (Table 3). There was an increased incidence of SC-ABMR during the 1st year in DSA+ vs. DSA- patients (4% vs. 0%, *p* < 0.001), though overall occurrence was rare (0.8%) (Table 3). There were seven cases of SC-ABMR in six recipients (1 SC-ABMR alone, 3 with concurrent SCI, 3 with concurrent SC-TCMR).

**TABLE 3:** Summary of protocol biopsy findings during the 1st year post-transplant for study recipients who had a stable 1st year post-transplant course and had at least one protocol biopsy during the 1st year. Percentages are reflective of percentage of biopsies (not patients) falling within each category.

	Total (N = 515)	DSA- (N = 419)	DSA+ (N = 96)	*p*-value
Mean Acute Score Sum at 3 months (i + t + v + g)	1.7 (1.7)	1.6 (1.6)	1.9 (1.8)	0.26
Mean Acute Score Sum at 12 months (i + t + v + g)	2.1 (2.0)	2.1 (1.9)	2.5 (2.4)	0.16
Mean IFTA Score 3 months (ct + ci)	1.4 (1.1)	1.4 (1.1)	1.4 (0.9)	0.99
Mean IFTA Score 12 months (ct + ci)	2.0 (1.1)	2 (1.2)	2 (0.9)	0.78
Number of Protocol Biopsies during the 1st year	855 (100%)	696 (81%)	159 (19%)	
Biopsy Grade–Max during 1st Year				0.25
No Inflammation % (95% CI)	13 (11–17)	14 (11–18)	11 (5–19)	
Subclinical Inflammation % (95% CI)	56 (51–60)	55 (50–60)	58 (47–68)	
Subclinical TCMR % (95% CI)	31 (27–35)	31 (26–35)	31 (22–41)	
Biopsy Grade–3 months				0.91
No Inflammation % (95% CI)	27 (23–32)	28 (23–33)	26 (17–36)	
Subclinical Inflammation % (95% CI)	56 (51–60)	55 (50–60)	57 (46–67)	
Subclinical TCMR % (95% CI)	17 (13–20)	17 (13–21)	17 (10–27)	
Biopsy Grade–12 months				0.34
No Inflammation % (95% CI)	22 (18–26)	22 (18–27)	20 (12–32)	
Subclinical Inflammation % (95% CI)	52 (47–57)	52 (47–58)	52 (40–64)	
Subclinical TCMR % (95% CI)	26 (22–30)	26 (21–31)	28 (18–39)	
Subclinical ABMR				
Anytime % (95% CI)	0.8 (0.3–2)	0	4 (2–9)	< 0.001
3 months % (95% CI)	0.9 (0.2–2)	0	5 (1–11)	< 0.001
12 months % (95% CI)	0.8 (0.1–2)	0	4 (1–12)	< 0.001
Type of Subclinical ABMR				
Sub-Clinical ABMR Alone % (95% CI)	0.1 (0–0.7)	0	0.6 (0–4)	**0.02**
Sub-Clinical ABMR + SCI % (95% CI)	0.4 (0.1–1)	0	2 (0.4–5)	< 0.001
Sub-Clinical ABMR + SC-TCMR % (95% CI)	0.4 (0.1–1)	0	2 (0.4–5)	< 0.001

Bold values considered statistically significant with *p*-value < 0.05.

Abbreviations are as follows: IFTA, interstitial fibrosis tubular atrophy; CI, Confidence interval; TCMR, T cell mediated rejection; ABMR, antibody mediated rejection; SCI, subclinical inflammation.

### For-Cause Biopsy Findings Beyond 1st Year

**FIGURE 2:**
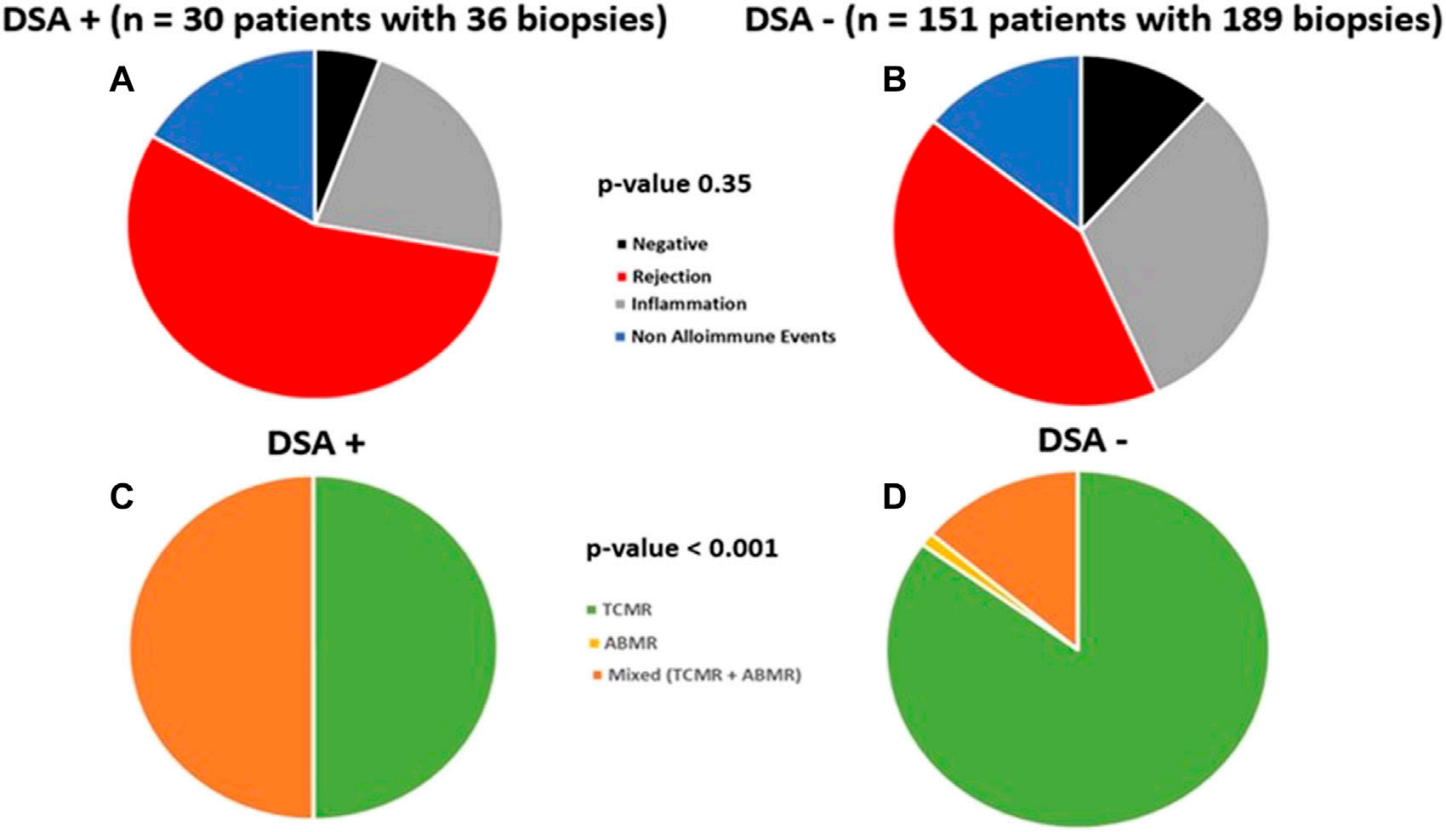
The distribution of for-cause biopsies performed beyond 1 year (*n* = 225, 36 in DSA+ vs. 189 in DSA-) are shown below. A total of 181 patients (25% total) received a for-cause biopsy beyond 1 year and this was similar among the groups (*n* = 30 in DSA+ [23%] vs. *n* = 151 in DSA- [25%], *p* = 0.62). The frequency of for-cause biopsies beyond 1 year demonstrating clinical rejection, clinical inflammation, and non-alloimmune events was similar among DSA+ and DSA-patients (*p* = 0.35, **(A,B)**). However, the type of rejection seen on late for-cause biopsies was different among the groups **(C,D)** as the frequency of biopsies in DSA+ patients trended towards more mixed rejection (50% [95% CI 27–73%] vs. 14% [95% CI 7–23%], *p* = 0.07) and less TCMR (50% [95% CI 27–73%] vs. 85% [95% CI 75–92%], *p* < 0.001) compared to DSA- patients.

DSA+ patients had similar proportion of for-cause biopsies beyond 1st year vs. DSA- patients (23% [30 patients, 36 biopsies] vs. 25% [151 patients, 189 biopsies], *p* = 0.62, Table 4). The distribution of biopsy findings was similar between DSA+ and DSA- cohorts (*p* = 0.35, Figures 2A,B), including rates of overall clinical rejection (56% vs. 42%, *p* = 0.14). Clinical TCMR was lower among DSA+ patients (28% vs. 36%, *p* < 0.001), though severity of TCMR was similar (Table 4). The distribution of type rejection was different (*p* < 0.001) and favored more mixed rejection (ABMR + TCMR) in DSA+ patients (Figures 2C,D). Lastly, distribution of non-alloimmune events on for-cause biopsies was similar (16% vs. 14%, *p* = 0.71, Table 4).

**TABLE 4:** Summary of for-cause kidney biopsies performed beyond 1 year for patients who had a stable 1st year post-transplant course. Patients were grouped by whether Donor Specific Antibody (DSA) was detected during the 1st year post-transplant. Percentages are reflective of percentage of biopsies (not patients) falling within each category.

	Total (N = 736)	DSA- (N = 605)	DSA+ (N = 131)	*p*-value
% Patients undergoing for-cause biopsy beyond 1 year	25 (*n* = 181)	25 (*n* = 151)	23 (*n* = 30)	0.62
Number of for-cause biopsies performed beyond 1 year	225 (100%)	189 (84%)	36 (16%)	
Distribution of biopsies				0.35
Negative % (95% CI)	11 (7–15)	12 (7–17)	6 (1–19)	
Clinical inflammation % (95% CI)	30 (24–37)	32 (25–39)	22 (10–39)	
Clinical rejection % (95% CI)	44 (38–51)	42 (35–50)	56 (38–72)	
Non alloimmune events % (95% CI)	15 (10–20)	14 (10–20)	16 (6–33)	
Clinical inflammation % (95% CI)	30 (24–37)	32 (25–39)	22 (10–39)	0.25
Minimal inflammation % (95% CI)	6 (4–11)	8 (4–12)	3 (0.1–15)	0.49
Banff borderline changes % (95% CI)	24 (18–30)	24 (18–31)	19 (8–36)	0.49
Clinical rejection % (95% CI)	44 (38–51)	42 (35–50)	56 (38–72)	0.14
TCMR % (95% CI)	35 (28–41)	36 (29–43)	28 (14–45)	< 0.001
1A % (95% CI)	24 (18–30)	25 (19–32)	17 (6–33)	0.56
1B % (95% CI)	11 (7–15)	11 (7–16)	11 (3–26)	0.50
≥2A %	0.4 (0–3)	0.5 (0–3)	0 (0–10)	0.70
Mixed (associated TCMR grade) % (95% CI)	9 (6–14)	6 (3–10)	28 (14–45)	0.07
1A % (95% CI)	4 (2–7)	2 (0.6–5)	11 (3–26)	0.87
1B % (95% CI)	4 (2–8)	2 (0.6–5)	14 (5–30)	0.53
≥2A % (95% CI)	1 (0.6–5)	2 (0.3–5)	3 (0.1–15)	0.31
ABMR alone %	0.4 (0–3)	0.5 (0–3)	0 (0–10)	0.66
Non alloimmune events % (95% CI)	15 (10–20)	14 (10–20)	16 (6–33)	0.71
UTI % (95% CI)	2 (0.5–5)	0.5 (0–3)	8 (2–22)	**0.002**
BK virus nephropathy % (95% CI)	7 (4–11)	7 (4–11)	6 (0.7–19)	0.51
Acute tubular injury % (95% CI)	3 (2–7)	4 (2–7)	2 (0.1–15)	0.63
Glomerulonephritis % (95% CI)	2 (0.5–5)	2 (0.5–5)	0 (0–10)	0.31
Oxalate nephropathy % (95% CI)	1 (0.1–3)	1 (0.1–4)	0 (0–10)	0.49

Bold values considered statistically significant with *p*-value < 0.05.

### Kidney Function

**FIGURE 3:**
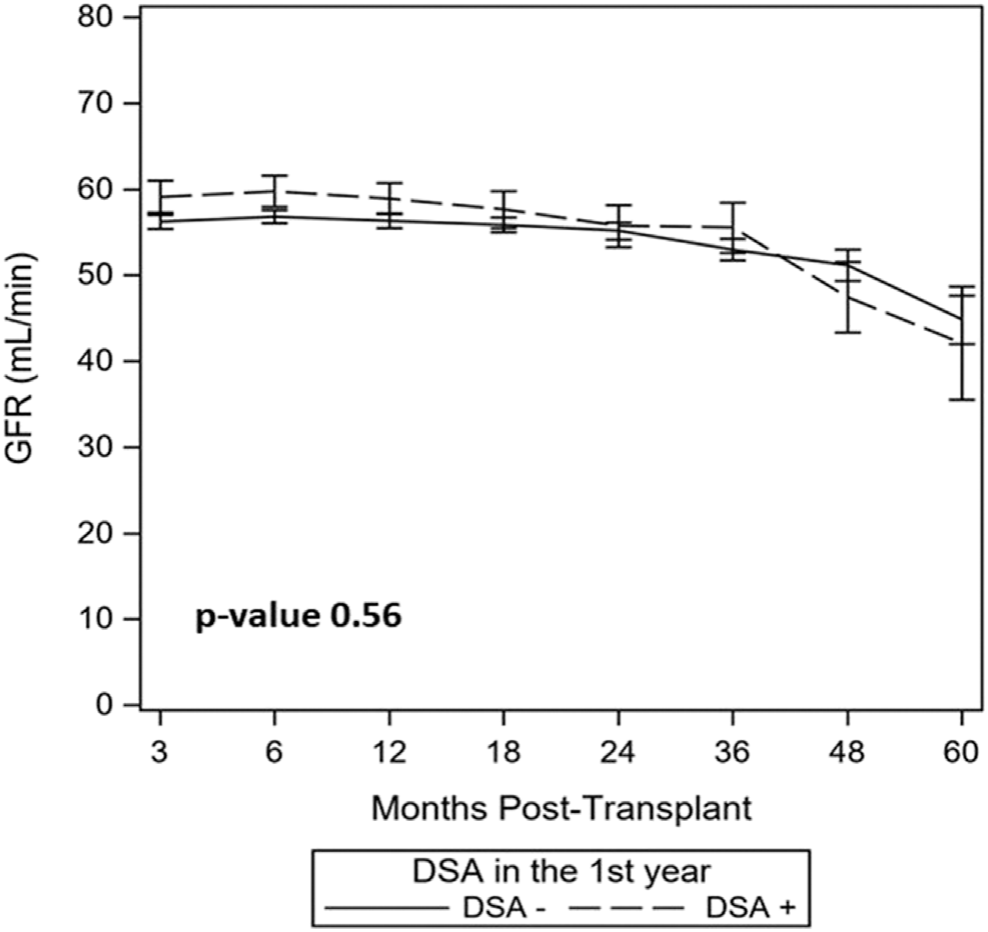
Similar kidney function was seen over study follow up in DSA+ and DSA-kidney transplant recipients with a stable 1st year post-transplant course. A linear mixed model with estimated glomerular filtration rates (GFR) over the study follow up period demonstrating similar GFR between DSA+ and DSA- patients over time (54.8 vs. 53.8 ml/min/1.73 m^2^, *p* = 0.56) is shown.

Using a linear mixed model, DSA+ and DSA- groups had similar eGFR over study period (54.8 vs. 53.8 ml/min/1.73 m^2^, *p* = 0.56, Figure 3). Subgroup exploratory analysis in those patients with protocol biopsy revealed eGFR was similar among DSA+ vs. DSA- patients when stratified by 1st year subclinical events (NI, SCI, SC-TCMR), albeit there was slightly increased eGFR for DSA+ with SCI vs. DSA-with SCI patients (*p* = 0.02, 61 ml/min vs. 54 ml/min, Supplementary Figure S1).

### Patient and Graft Survival

**FIGURE 4:**
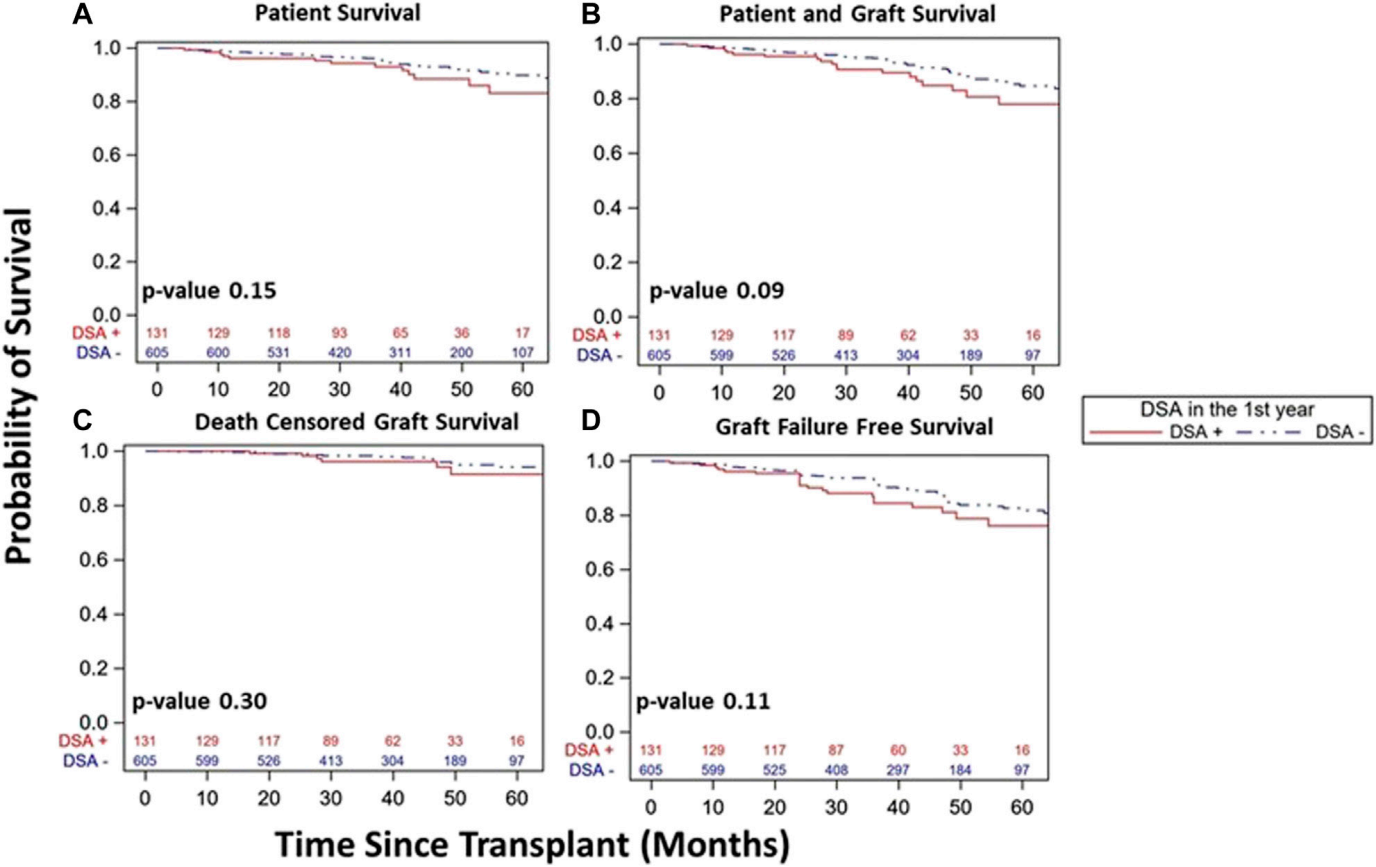
Similar kidney transplant and patient survival was seen over study follow up in DSA+ and DSA- kidney transplant recipients with a stable 1st year post-transplant course. Kaplan-Meier survival curves demonstrated similar patient survival (**(A)**, 83% [95% CI 71–91%]) vs. 90% [95% CI 86–93%], *p* = 0.15), patient and graft survival (**(B)**, 78% [95% CI 65–86%] vs. 85% [95% CI 80–88%], *p* = 0.09), death censored graft survival (**(C)**, 91% [95% CI 80–96%] vs. 94% [95% CI 90–97%], *p* = 0.30), and graft failure free survival (**(D)**, 76% [95% CI 64–85%] vs. 82% [95% CI 77–87%], *p* = 0.11) among DSA+ and DSA-patients over study period follow up.

**FIGURE 5:**
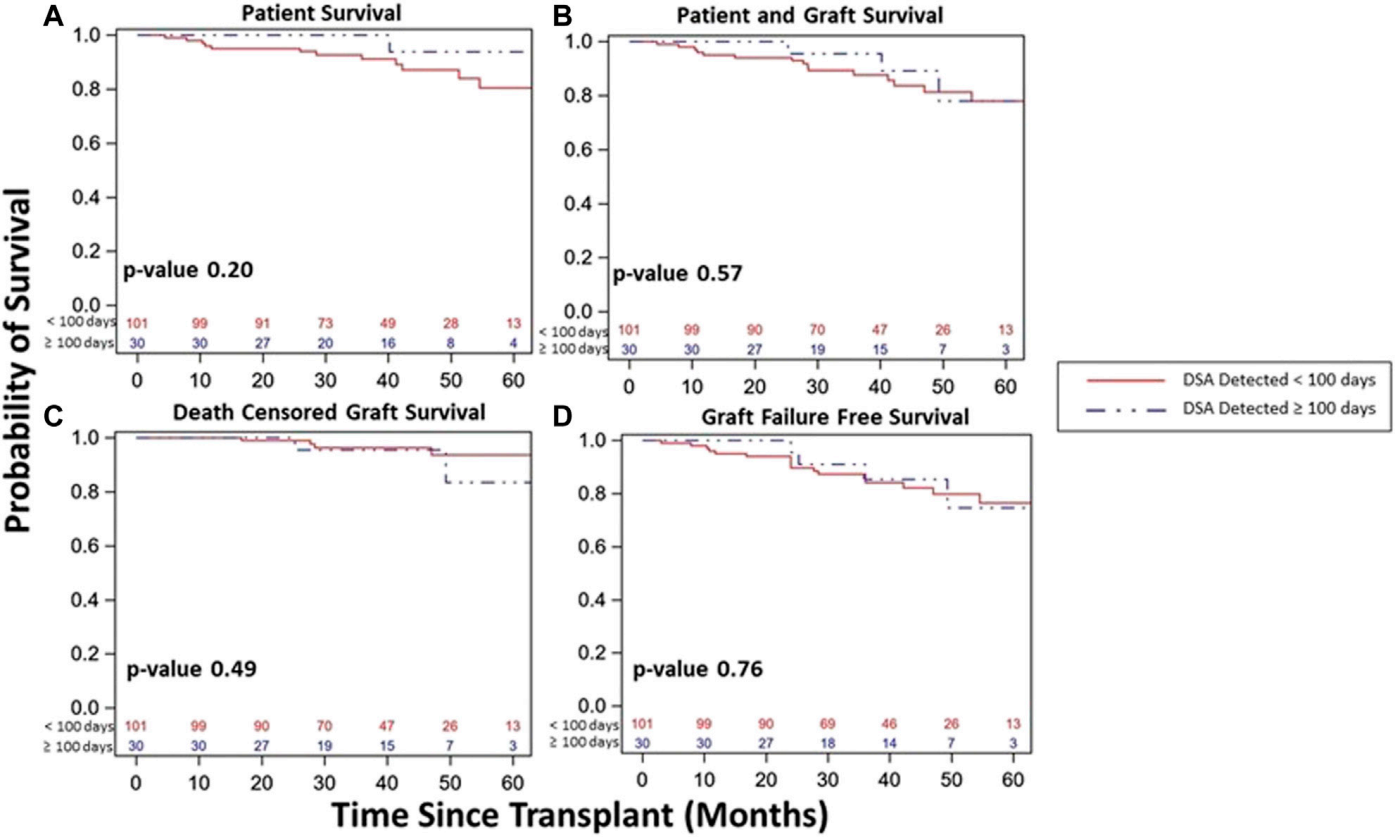
Kidney transplant and patient survival for patients with DSA detected and a stable clinical course during the 1st year post kidney transplant based on timing of DSA detection status within the 1st year post-transplant (<100 days vs. ≥100 days). Kaplan-Meier survival curves demonstrating patient survival (**(A)**, 80% vs. 94%, *p* = 0.20), patient and graft survival (**(B)**, 78% vs. 78%, *p* = 0.57), death censored graft survival (**(C)**, 94% vs. 85%, *p* = 0.49), and graft failure free survival (**(D)**, 76% vs. 75%, *p* = 0.76) over study period follow up among patients with DSA detected and a stable clinical course during the 1st year post-transplant based on timing of DSA detection.

**FIGURE 6:**
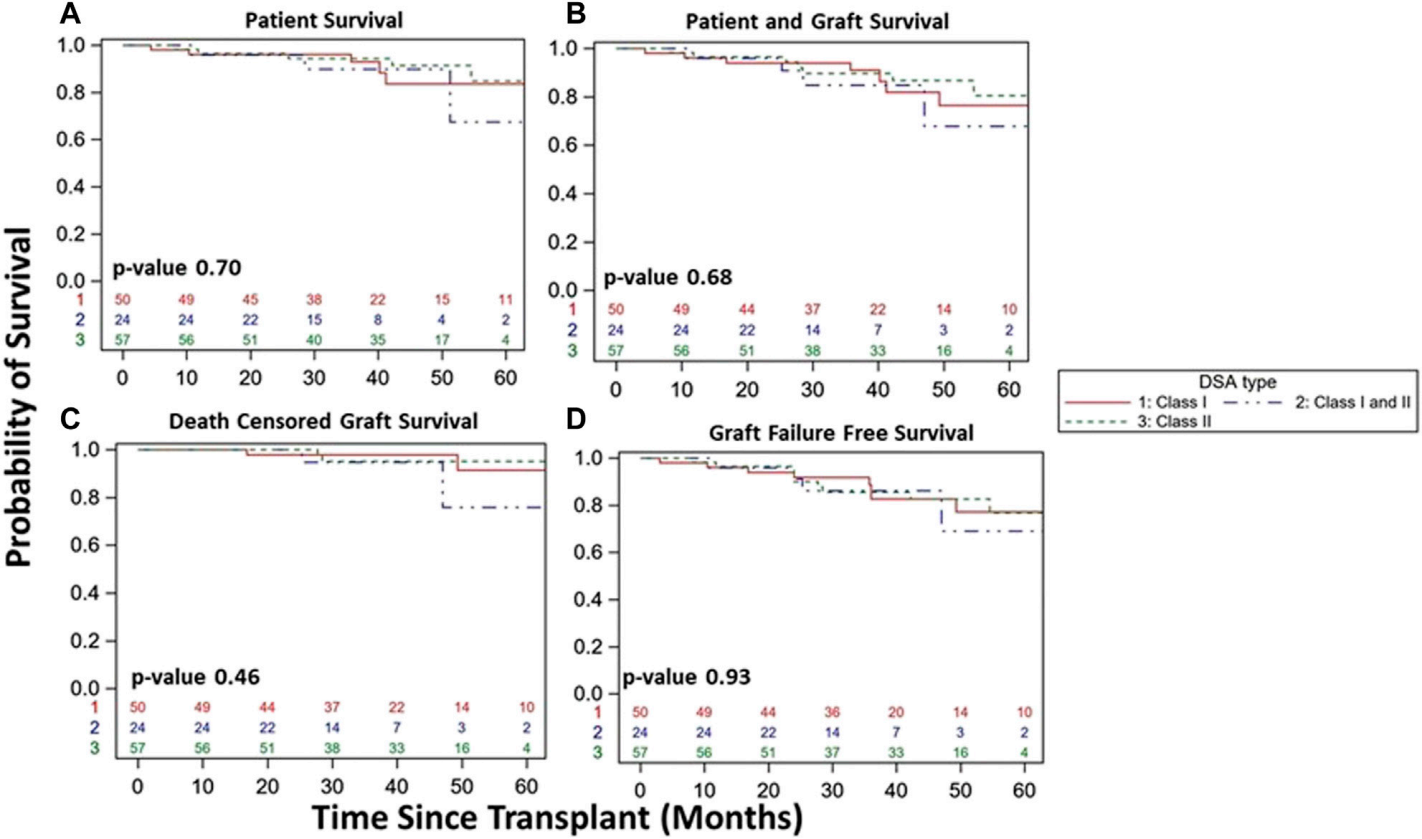
Kidney transplant and patient survival for patients with DSA detected and stable clinical course during the 1st year post kidney transplant based on DSA class detected (Class I vs. Class I/II vs. Class II). Kaplan-Meier survival curves demonstrating patient survival (**(A)**, 84% vs. 67% vs. 85%, *p* = 0.70), patient and graft survival (**(B)**, 76% vs. 68% vs. 81%, *p* = 0.68), death censored graft survival (**(C)**, 91% vs. 76% vs. 95%, *p* = 0.46), and graft failure free survival (**(D)**, 77% vs. 69% vs. 77%, *p* = 0.93) over study period follow up among DSA+ patients with a stable clinical course during the 1st year post-transplant.

Overall, DSA+ patients had similar patient survival (83% vs. 90%, *p* = 0.15, Figure 4A), combined patient and graft survival (78% vs. 85%, *p* = 0.09, Figure 4B), DCGS (91% vs. 94%, *p* = 0.30, Figure 4C), and graft failure free survival (76% vs. 82%, *p* = 0.11, Figure 4D) vs. DSA- patients. Among DSA+ patients, survival was similar whether based on timing of detection (Figure 5) or DSA class (Figure 6). We also assessed survival stratified by protocol biopsy status. First, those with protocol biopsy had better patient survival (*p* = 0.004), combined patient and graft survival (*p* = 0.02), and graft failure free survival compared to those without protocol biopsy (*p* = 0.045), but not DCGS (*p* = 0.68) (Supplementary Figure S2). Among those without protocol biopsy, DSA+ patients had similar survival vs. DSA- patients (Supplementary Figure S3). Conversely, among those with at least one protocol biopsy, DSA+ patients had decreased patient (*p* = 0.04), patient and graft (*p* = 0.02), graft failure free survival (*p* = 0.05), but not DCGS (*p* = 0.13) compared to DSA-patients (Supplementary Figure S4).

### Multivariate Analysis for Graft Failure

Given a trend towards worse graft failure free survival in DSA+ patients, specifically those who had a protocol biopsy, we performed a backwards cox regression multivariate analysis that found recipient age (1.03, 95% CI 1.01–1.05, *p* = 0.01) and DSA detection within 1st year (1.91, 95% CI 1.03–3.55, *p* = 0.04) as independent predictors for graft failure among those who had a protocol biopsy (Table 5).

**TABLE 5:** Adjusted multivariate analysis showing risk factors for developing graft failure among those with at least one protocol biopsy during the 1st year (*n* = 515).

	Hazard ratio (with 95% CI)	*p*-value
DSA during 1st year	1.91 (1.03–3.55)	**0.04**
Recipient Age	1.03 (1.01–1.05)	**0.01**
Subclinical TCMR during 1st year	1.14 (0.48–2.70)	0.76
Subclinical Inflammation during 1st year	0.90 (0.41–1.98)	0.80

Bold values considered statistically significant with *p*-value < 0.05.

Adjusted multivariate model was adjusted for recipient age, donor type (Living donor vs. Deceased donor), PRA I/II ≥ 90%, cPRA ≥ 90%, DGF, and SC-I/SC-TCMR, using a backward selection Cox Regression Model.

### Unstable Patients

**FIGURE 7:**
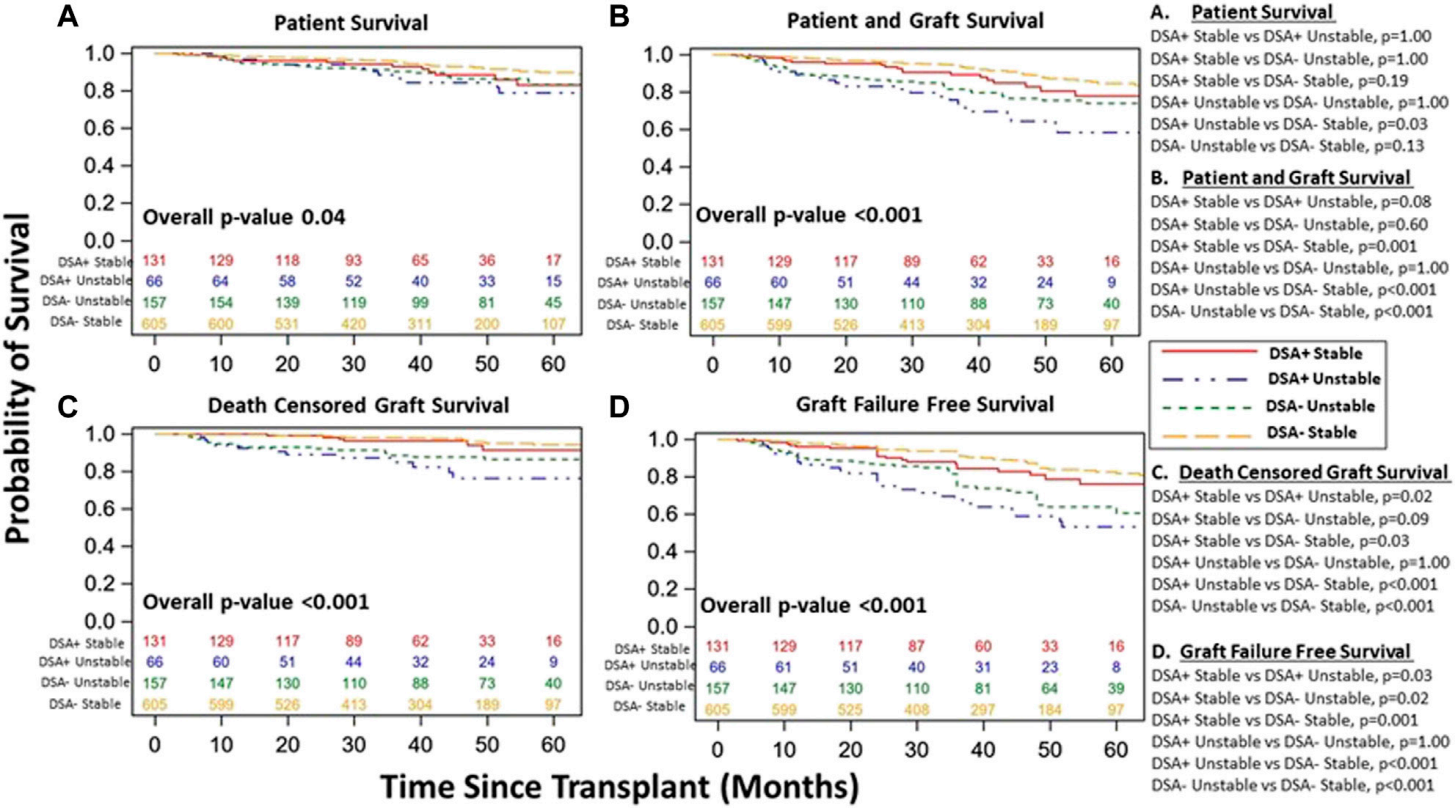
Kidney transplant and patient survival for all patients (included and excluded patients) grouped based on DSA detection status (DSA+ versus DSA-) and clinical course (stable versus unstable) during the 1st year post kidney transplant. Kaplan-Meier survival curves demonstrating patient survival (**(A)**, DSA+ Stable 83% [95% CI 71–91%]) vs. DSA- Stable 90% [95% CI 86–93%] vs. DSA+ Unstable 76% [95% CI 61–86%] vs. DSA- Unstable 86% [95% CI 79–91%], *p* = 0.04), patient and graft survival (**(B)**, DSA+ Stable 78% [95% CI 65–86%] vs. DSA- Stable 85% [95% CI 80–88%] vs. DSA+ Unstable 58% [95% CI 43–71%] vs. DSA- Unstable 74% [95% CI 65–81%], *p* < 0.001), death censored graft survival (**(C)**, DSA+ Stable 91% [95% CI 80–96%] vs. DSA- Unstable 94% [95% CI 90–97%] vs. DSA+ Unstable 76% [95% CI 61–86%] vs. DSA- Unstable 86% [95% CI 79–91%], *p* < 0.001), and Graft Failure free survival (**(D)**, DSA+ Stable 76% [95% CI 64–85%] vs. DSA- Stable 82% [95% CI 77–87%] vs. DSA+ Unstable 53% [95% CI 38–66%] vs. DSA- Unstable 61% [95% CI 51–69%], *p* < 0.001) over study period follow up among all patients. The individualized comparisons for **(A–D)** are provided.

Though excluded from our primary cohort analysis, we did note increased DSA within 1st year in unstable vs. DSA+ stable patients (30% vs. 18%, *p* < 0.001). We explored demographic differences among stable and unstable cohorts based on DSA status (Supplementary Tables S2, S3) and DSA characteristics among DSA+ patients (unstable vs. stable) (Supplementary Table S4). Interestingly, DSA- unstable patients received kidney transplants with higher KDPI and had more DGF than DSA- stable patients (Supplementary Table S2). Further, DSA+ unstable patients were younger (46 vs. 51, *p* = 0.02) with increased overall number of DSA tests, overall number of positive class II tests, and a trend towards more combined Class I and Class II DSA detection vs. DSA+ stable patients, though the timing of 1st positive test (Class I/II) was similar for DSA+ unstable vs. DSA+ stable patients (Supplementary Tables S3, S4). Patient survival, patient and graft survival, DCGS, and graft failure free survival were similar among DSA+ vs. DSA- unstable patients (Supplementary Figure S5). However, when all four groups were included, there was significant differences in survival among the four groups as the unstable cohort had inferior survival overall, particularly the DSA+ unstable group (Figure 7). Interestingly, when including entire population (both stable and unstable), DSA+ stable patients did have inferior patient/graft survival (*p* = 0.001), DCGS (*p* = 0.03), and graft failure free survival (*p* = 0.001) vs. DSA-stable patients (Figure 7).

## Discussion

While post-transplant DSA detection has been associated with inferior outcomes, not all patients with post-transplant DSA fare poorly. Thus, whether early post-transplant DSA screening should be widely used in stable patients for risk stratification remains unclear. To address the impact of DSA detection as an early post-transplant screening tool, we assessed DSA detection on screening testing in stable patients for associations with key clinical events.

In a cohort of 736 patients with a stable 1st year course, DSA detection was not associated with inferior function or survival. Among those who had a protocol biopsy, DSA was associated with graft failure on multivariate analysis and increased early incidence of SC-ABMR. Specifically, DSA+ patients had increased SC-ABMR, but did not have increased SCI, SC-TCMR, or early chronicity (ie IFTA). Similarly, previous studies displayed increased subclinical rejection (mostly ABMR) with protocol biopsies performed for DSA detection on screening without graft dysfunction, though those often were later (beyond 1 year) and again not all patients had rejection (8,26–30). Still, Loupy et al. noted early SC-ABMR may impact long-term outcomes (31). While data on treatment of early SC-ABMR is limited, treatment of late SC-ABMR (∼55 months) may be effective, and thus diagnosing early SC-ABMR may be valuable (32). The reported incidence of SC-ABMR has been variable (∼26–51%), which is likely related to DSA and biopsy timing (events beyond 1 year), and differing study cohorts and designs (biopsy for DSA detection without graft dysfunction) (8, 27–30). SC-ABMR was a rare overall event in our low risk cohort likely due to DSA detection timing (within 1 year) and biopsy approach (no protocol biopsies for isolated DSA detection), though similar with the rate of ABMR (3.7%), albeit clinical, within 1st year reported by Adebiyi et al in their cohort with pre-transplant DSA that had a negative FC (33).

Beyond the 1st year, DSA+ patients had similar kidney function and rates of clinical rejection, though distribution was towards more mixed rejection (ABMR + TCMR). Comparably, Bartel et al. demonstrated post-transplant anti-HLA antibody detection with a stable 1st year course was not associated with worse eGFR at 5 years (34). Later, Cooper et al. showed patients with DSA had more clinical rejection (TCMR and/or ABMR), though intermediate (up to 24 months) outcomes (eGFR, graft survival) were similar for those with DSA without clinical rejection and DSA detected on screening compared to those without DSA detection (9). Likewise, Devos et al. reported DSA detection was associated with increased clinical rejection and worse DCGS at intermediate follow up (∼31 months), but there was no difference in graft survival or function for those with DSA without clinical rejection (35). While we recognize our DSA+ cohort as having DSA that was newly detected post-transplant, pre-existing DSA prior to transplant is possible given early DSA detection, which may explain similar survival outcomes as Aubert et al noted ABMR due to preexisting DSA occurs earlier than ABMR due to *de novo* DSA with better graft survival (36). Further, Adebiyi et al demonstrated a trend towards diminished DCGS in those with pre-transplant DSA, FC negative with post-transplant DSA versus those with no pre-transplant DSA or those with pre-transplant DSA but no post-transplant DSA (33). Likewise, in our study, DSA+ patients had similar survival (patient, DCGS) and function, though 1st year screening DSA detection was an independent predictor of graft failure on multivariate analysis only among those with protocol biopsy.

Previous studies were limited by smaller sample size and mixed testing indication (DSA and biopsy). For-cause DSA testing at time of dysfunction or for-cause biopsy biases towards adverse outcomes and is a different context than DSA screening testing in stable patients. Pediatric literature has demonstrated the reasoning (screening vs. for-cause) for DSA and biopsy testing matters in understanding DSA as a decision tool (37). Now, in a large adult cohort with clear testing indication, we demonstrate DSA detection on screening testing during the 1st year in stable kidney transplant patients was associated with increased SC-ABMR and was an independent predictor for graft failure among those who had a protocol biopsy, but not associated overall with inferior function or survival.

The strengths of our study include our large cohort with detailed histological (including protocol and for-cause biopsies) and clinical information. Further, our design to differentiate the reason for DSA and biopsy testing allowed better analysis of DSA as a screening tool in stable patients. Additionally, our study provided important information regarding the timing, type, and persistence of screening DSA in stable patients as well how these DSA and demographic characteristics for stable patients differed from those who had an unstable 1st year clinical course.

We acknowledge our study has limitations. First, our study is a single center study without an external validation cohort, which may limit broad applicability. Second, while our study was primarily focused on graft outcomes beyond the 1st year, we included the entire study period from the time of transplant for both our linear mixed eGFR model analysis and our Kaplan Meier survival analysis. Thus, we acknowledge results within the 1st year, while appearing similar, should be interpreted with caution as our study groups were defined by DSA detection at the end of the 1st year post-transplantation. Additionally, despite a large sample size, we could not perform adequate subgroup analysis among DSA+ patients for subclinical and clinical events to identify subgroups at higher risk who may benefit from more intense screening. Also, we did not have full information about HLA eplet mismatch load or about all DSA characteristics (MFI, titer, specificity, and complement binding), which both may allow better risk stratification, though limitations with MFI have been previously noted (13–15, 33, 38). Further, follow up period may be insufficient to detect true long-term differences in graft survival, though knowing this limitation, we did assess surrogate markers such as histology and eGFR. We also recognize the temporal relationship of DSA with both subclinical and clinical events, which we did not examine, though timing of first DSA detection was similar for stable vs. unstable groups. Also, previous studies suggest that not all DSA detection may precede rejection and this distinction may not impact associations with later events (5). As previously acknowledged, prior sera (>30 days prior to transplant) was not analyzed for historical DSA and we recognize our MFI cut off value of 1,000 may have missed weak DSA (MFI <1,000) at the time of transplant. Thus, early DSA detected in our study may have been pre-formed, which may be different from *de novo* DSA, though we did assess survival outcomes based on timing of DSA detection, which was not different. More, we did not assess medication non-adherence, which has been linked with DSA detection and poor outcomes, though this has been previously explored (5, 39, 40). We also recognize that DSA detection on screening may have influenced optimization of immunosuppression, which we could not account for, and this itself may have affected outcomes. Lastly, we recognize that our study cohort was heterogenous as ∼30% were without a protocol biopsy and this limited evaluation of subclinical events for all patients. However, we performed additional analysis assessing differences (demographics, survival) between those who did and did not receive protocol biopsies to give a more complete picture of our study cohort. Again, while heterogenous, our study represented an actual clinical practice where DSA screening would be used.

Nonetheless, we report key findings regarding early DSA screening among stable kidney transplant patients. Overall, DSA+ patients had similar function and survival vs. DSA- patients. In those with a protocol biopsy, DSA+ patients had increased incidence of SC-ABMR, with rare events overall, and similar incidence of SCI/SC-TCMR. Still, DSA detection was independently associated with graft failure among those who had a protocol biopsy. Lastly, DSA+ patients had similar incidence of clinical rejection on for-cause biopsies after 1 year vs. DSA- patients, though rejection was more mixed (ABMR + TCMR) in DSA+ patients. Additional studies involving multiple centers with an increased study population (especially given differences seen when including both stable and unstable cohorts, including between DSA+ vs. DSA- stable patients) and longer follow up may allow for more definitive evidence regarding the utility of DSA as an early post-transplant screening tool. More importantly, these types of studies may help definitively identify those patients who will benefit the most from intense early screening. Still, with our findings, a more targeted screening approach may increase the impact of DSA screening in stable patients and allow for more tailored medicine. Specifically, potential targeted approaches may include more intense screening in highly sensitized patients and/or those with increased DR mismatches, less intense screening in those with competing risk factors for graft loss (possibly similar to the non-protocol biopsy group), and/or using early intensive DSA screening within 6 months in all stable patients (majority of DSA+ tests were noted by this time, both for the stable and unstable cohorts) to guide further testing. Additionally, given association with subsequent DSA development, the consideration of eplet mismatches to guide early post-transplant screening may also increase impact (41–43). Regardless, with focus on high value care, cost effectiveness for DSA as a screening tool must be assessed as well given a previous study estimated the cost of annual DSA screening at ∼$480/year (range $300–1,000) and more recently, a single DSA screening test (combined for both Class I and II) was recently estimated at ∼$680 based on recent United States Medicare data, both of which highlight the need for more targeted screening in those low risk patients with stable kidney function (44–45). Lastly, the context and reason for DSA testing matters and should be clearly delineated in further studies as DSA for-cause testing assists in decision making when faced with renal dysfunction or supplements abnormal pathology whereas DSA screening testing in those with stable function may identify those at increased risk but impact may be blunted when widely used.

In conclusion, DSA detected on screening in stable 1st year kidney transplant patients was independently associated with graft failure on multivariate analysis, however this was only true among patients who underwent at least one protocol biopsy.

## Data Availability

The data that support the findings of this study are available on request from the corresponding author. The data are not publicly available due to privacy considerations.

## Abbreviations

ABMR, antibody mediated rejection; BBC, banff borderline changes; CIT, cold ischemia time; cPRA, calculated panel reactive antibody; DCGS, death censored graft loss; DD, deceased donor; DGF, delayed graft function; DSA, donor specific antibody; eGFR, estimated glomerular filtration rate; FC, flow crossmatch; ESKD, end stage kidney disease; HLA, human leukocyte antigen; IFTA, interstitial fibrosis and tubular atrophy; IRB, institutional review board; KDPI, kidney donor prognostic index; LD, living donor; MFI, mean fluorescence intensity; MI, minimal inflammation; NI, no inflammation; PRA, panel reactive antibody; SCI, subclinical inflammation; SC-TCMR, subclinical T cell mediated rejection; SC-ABMR, subclinical antibody mediated rejection; TCMR, T cell mediated rejection.
