# Supplementary material for: The Clinical Impact of Anti-HLA Donor Specific Antibody Detection Through First Year Screening on Stable Kidney Transplant Recipients
Source: Transpl Int. 2022 Mar 17;35:10094. doi: 10.3389/ti.2022.10094 (PMC8967948; doi:10.3389/ti.2022.10094)

**Table S1.** Characteristics of recipients who had a stable 1^st^ year post-transplant course based on whether they had at least one protocol biopsy during the 1^st^ year versus no protocol biopsy during the 1^st^ year.

|  | **Total (N =736)** | **No Protocol Biopsy (N = 221)** | **Protocol Biopsy (N = 515)** | **p-value** |
| --- | --- | --- | --- | --- |
| **Recipient Age (years, Mean/SD)** | 52 (14) | 53 (13) | 52 (14) | 0.29 |
| **Recipient Gender (% Male)** | 59 | 59 | 58 | 0.83 |
| **Recipient Race (% Caucasian)** | 76 | 80 | 75 | 0.29 |
| **Body Mass Index at Transplant (Kg/m2, Mean/SD)** | 28.7 (5.7) | 28.2 (5.6) | 28.9 (5.8) | 0.16 |
| **Preemptive Transplant (%)** | 19 | 14 | 21 | **0.03** |
| **Prior Kidney Transplant (%)** | 17 | 22 | 15 | **0.02** |
| **Any Prior Transplant (%)** | 25 | 32 | 21 | 0.08 |
| **Cause of End Stage Kidney Disease (%)** |  |  |  |  |
| Diabetes Mellitus | 22 | 32 | 19 | **<0.001** |
| Hypertension | 18 | 16 | 19 | 0.38 |
| Polycystic Kidney Disease | 11 | 9 | 13 | 0.12 |
| Glomerulonephritis | 4 | 4 | 5 | 0.40 |
| Other/Unknown | 45 | 39 | 44 | 0.15 |
| **Deceased Donor (%)** | 68 | 77 | 65 | **0.002** |
| **Donor Age (years, Mean/SD)** | 40 (14) | 37 (15) | 41 (14) | **<0.001** |
| **Donor Gender (% Male)** | 55 | 56 | 55 | 0.70 |
| **Donor Race (% Caucasian)** | 89 | 89 | 88 | 0.84 |
| **Cold Ischemia Time (minutes, Median/IQR)** | 506 (88-792) | 540 (320-820) | 482 (77-778) | **0.02** |
| **Kidney Donor Prognostic Index (%, Mean/SD)** | 42 (25) | 41 (25) | 43 (26) | 0.37 |
| **% with Panel Reactive Antibody Class I ≥ 90%** | 18 | 23 | 16 | **0.02** |
| **% with Panel Reactive Antibody Class II ≥ 90%** | 20 | 26 | 18 | **0.006** |
| **Total Mismatches (Median/IQR)** | 4 (3-5) | 4 (3-5) | 4 (3-5) | 0.76 |
| **DR Mismatches (Median/IQR)** | 1 (1-2) | 1 (1-2) | 1 (1-2) | 0.75 |
| **Cytomegalovirus D+/R- (%)** | 21 | 17 | 22 | 0.08 |
| **Epstein-Barr Virus D+/R- (%)** | 5 | 4 | 6 | 0.37 |
| **Delayed Graft Function (%)** | 16 | 17 | 16 | 0.68 |
| **Donor Specific Antibody detected during 1^st^ year (%)** | 18 | 16 | 19 | 0.36 |
| **Donor Specific Antibody detected after 1^st^ year (%)** | 15 | 16 | 15 | 0.57 |
| **Median Follow Up (days, Median/IQR)** | 1199 (808-1640) | 1151 (759-1613) | 1205 (829-1646) | 0.30 |

**Table S2.** Characteristics of recipients without DSA detection during 1^st^ year post-transplant course based on whether they had stable 1^st^ year course versus unstable 1^st^ year course.

|  | **Total (N=762)** | **DSA- Stable**  **(N=605)** | **DSA- Unstable (N=157)** | **p-value** |
| --- | --- | --- | --- | --- |
| **Recipient Age (years, Mean/SD)** | 53 (14) | 53 (14) | 52 (14) | 0.47 |
| **Recipient Gender (% Male)** | 61 | 60 | 66 | 0.20 |
| **Recipient Race (% Caucasian)** | 77 | 78 | 71 | 0.19 |
| **Body Mass Index at Transplant (Kg/m2, Mean/SD)** | 28.5 (5.7) | 28.5 (5.7) | 28.8 (5.8) | 0.60 |
| **Preemptive Transplant (%)** | 20 | 20 | 19 | 0.70 |
| **Prior Kidney Transplant (%)** | 16 | 16 | 15 | 0.74 |
| **Any Prior Transplant (%)** | 25 | 25 | 25 | 0.86 |
| **Cause of End Stage Kidney Disease (%)** |  |  |  |  |
| Diabetes Mellitus | 22 | 22 | 24 | 0.55 |
| Hypertension | 18 | 19 | 15 | 0.28 |
| Polycystic Kidney Disease | 11 | 11 | 8 | 0.26 |
| Glomerulonephritis | 5 | 5 | 4 | 0.55 |
| Other/Unknown | 44 | 43 | 49 | 0.43 |
| **Deceased Donor (%)** | 66 | 67 | 66 | 0.81 |
| **Donor Age (years, Mean/SD)** | 41 (14) | 40 (14) | 44 (13) | **0.003** |
| **Donor Gender (% Male)** | 55 | 55 | 53 | 0.65 |
| **Donor Race (% Caucasian)** | 89 | 89 | 88 | 0.71 |
| **Cold Ischemia Time (minutes, Median/IQR)** | 492 (85-783) | 497 (85-792) | 480 (93-736) | 0.88 |
| **Kidney Donor Prognostic Index (%, Mean/SD)** | 44 (25) | 43 (25) | 51 (24) | **0.003** |
| **% with Panel Reactive Antibody Class I ≥ 90%** | 16 | 15 | 17 | 0.73 |
| **% with Panel Reactive Antibody Class II ≥ 90%** | 17 | 18 | 15 | 0.47 |
| **Total Mismatches (Median/IQR)** | 4 (3-5) | 4 (3-5) | 5 (3-5) | 0.10 |
| **DR Mismatches (Median/IQR)** | 1 (1-2) | 1 (1-2) | 1 (1-2) | 0.15 |
| **Cytomegalovirus D+/R- (%)** | 21 | 22 | 20 | 0.86 |
| **Epstein-Barr Virus D+/R- (%)** | 4 | 5 | 3 | 0.34 |
| **Delayed Graft Function (%)** | 18 | 16 | 29 | **<0.001** |
| **Donor Specific Antibody detected during 1^st^ year (%)** | 0 | 0 | 0 | n/a |
| **Donor Specific Antibody detected after 1^st^ year (%)** | 10 | 10 | 9 | 0.67 |
| **Median Follow Up (days, Median/IQR)** | 1213 (784-1672) | 1204 (805-1646) | 1305 (777-1806) | 0.20 |

**Table S3.** Characteristics of recipients with DSA detection during 1^st^ year post-transplant course based on whether they had stable 1^st^ year course versus unstable 1^st^ year course.

|  | **Total (N=197)** | **DSA+ Stable (N=131)** | **DSA+ Unstable (N=66)** | **p-value** |
| --- | --- | --- | --- | --- |
| **Recipient Age (years, Mean/SD)** | 49 (13) | 51 (12) | 46 (15) | **0.02** |
| **Recipient Gender (% Male)** | 56 | 53 | 62 | 0.21 |
| **Recipient Race (% Caucasian)** | 67 | 68 | 64 | 0.21 |
| **Body Mass Index at Transplant (Kg/m2, Mean/SD)** | 29.2 (5.7) | 29.6 (5.9) | 28.4 (5.4) | 0.18 |
| **Preemptive Transplant (%)** | 13 | 12 | 14 | 0.78 |
| **Prior Kidney Transplant (%)** | 22 | 21 | 23 | 0.83 |
| **Any Prior Transplant (%)** | 25 | 24 | 27 | 0.83 |
| **Cause of End Stage Kidney Disease (%)** |  |  |  |  |
| Diabetes Mellitus | 22 | 25 | 17 | 0.18 |
| Hypertension | 16 | 19 | 20 | 0.35 |
| Polycystic Kidney Disease | 11 | 11 | 11 | 0.86 |
| Glomerulonephritis | 4 | 5 | 5 | 0.69 |
| Other/Unknown | 47 | 43 | 47 | 0.29 |
| **Deceased Donor (%)** | 73 | 76 | 65 | 0.10 |
| **Donor Age (years, Mean/SD)** | 40 (14) | 39 (13) | 42 (15) | 0.22 |
| **Donor Gender (% Male)** | 54 | 56 | 52 | 0.58 |
| **Donor Race (% Caucasian)** | 88 | 86 | 94 | 0.20 |
| **Cold Ischemia Time (minutes, Median/IQR)** | 514 (93-766) | 544 (204-782) | 483 (58-735) | 0.15 |
| **Kidney Donor Prognostic Index (%, Mean/SD)** | 43 (25) | 41 (25) | 47 (24) | 0.17 |
| **% with Panel Reactive Antibody Class I ≥ 90%** | 34 | 30 | 41 | 0.12 |
| **% with Panel Reactive Antibody Class II ≥ 90%** | 34 | 31 | 38 | 0.36 |
| **Total Mismatches (Median/IQR)** | 5 (4-5) | 4 (4-5) | 5 (4-6) | 0.21 |
| **DR Mismatches (Median/IQR)** | 1 (1-2) | 1 (1-2) | 1 (1-2) | 0.22 |
| **Cytomegalovirus D+/R- (%)** | 20 | 16 | 29 | 0.09 |
| **Epstein-Barr Virus D+/R- (%)** | 7 | 7 | 6 | 0.99 |
| **Delayed Graft Function (%)** | 20 | 17 | 26 | 0.14 |
| **Donor Specific Antibody detected during 1^st^ year (%)** | 100 | 100 | 100 | n/a |
| **Donor Specific Antibody detected after 1^st^ year (%)** | 44 | 39 | 53 | 0.06 |

| **DSA Characteristic** | **Total = 197** | **DSA+ Stable (N = 131)** | **DSA+ Unstable (N=66)** | **P-Value** |
| --- | --- | --- | --- | --- |
| **# of Class I Tests during 1^st^ year*** | 8 (6-11) | 8 (6-10) | 10 (7-12) | **0.002** |
| **# of Class II Tests during 1^st^ year*** | 8 (6-11) | 8 (6-10) | 10 (7-13) | **0.002** |
| **# of + Class I Tests during 1^st^ year*** | 1 (0-3) | 1 (0-2) | 1 (0-4) | 0.08 |
| **# of + Class II Tests during 1^st^ year*** | 1 (0-4) | 1 (0-3) | 3 (1-6) | **0.01** |
| **Time to + Class I Test (days)*** | 39 (27-113) | 41 (30-108) | 33 (18-166) | 0.13 |
| **Time to + Class II Test (days)*** | 38 (29-177) | 38 (31-135) | 40 (26-217) | 0.35 |
| **Class I + % during 1^st^ year** | 59 | 57 | 65 | 0.24 |
| **Class II + % during 1^st^ year** | 64 | 62 | 68 | 0.38 |
| **Single Positive DSA (%)** | 46 | 46 | 46 | 0.96 |
| **Multiple Positive DSA (%)** | 63 | 60 | 68 | 0.28 |
| **DSA Type** |  |  |  | 0.06 |
| Class I (%) | 36 | 38 | 32 |  |
| Class II (%) | 41 | 44 | 35 |  |
| Class I & Class II (%) | 23 | 18 | 33 |  |

**Table S4.** Differences in Donor Specific Antibody (DSA) characteristics for DSA+ patients with a stable 1^st^ year post-transplant course versus DSA+ patients with an unstable 1^st^ year post-transplant course

*Median with IQ range

**Figure S1.** Kidney function based stratified by 1^st^ year renal allograft biopsy findings and DSA status. A linear mixed model with estimated glomerular filtration rates (GFR) over the study follow up period for those patients with at least 1 protocol biopsy during the 1^st^ year post transplant & stratified by their 1^st^ year protocol biopsy findings (max grade, **1A –** No Inflammation, **1B** – Subclinical Inflammation**, 1C** – Subclinical TCMR).


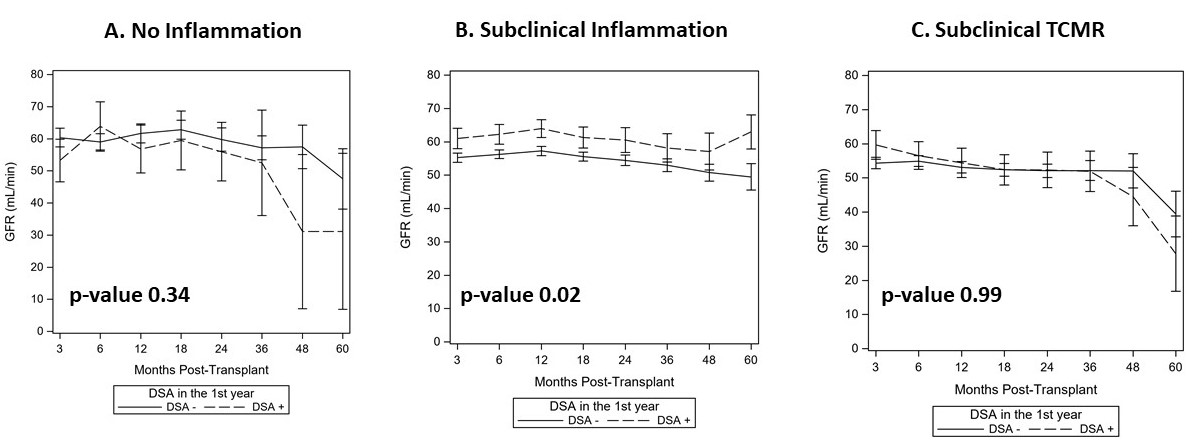


**Figure S2.** Kidney transplant and patient survival for all patients with stable clinical course during the 1^st^ year post kidney transplant based on whether they had at least one protocol biopsy versus no protocol biopsies during the 1^st^ year. Kaplan-Meier survival curves demonstrating patient survival (**A,** 91% vs 84%, p=0.04), patient and graft survival (**B,** 85% vs 79%, p=0.02), death censored graft survival (**C,** 94% vs 94%, p=0.68), and Graft Failure free survival (**D**, 82% vs 78%, p=0.045) over study period follow up among patients with stable clinical course during the 1^st^ year post-transplant based on protocol biopsy status.


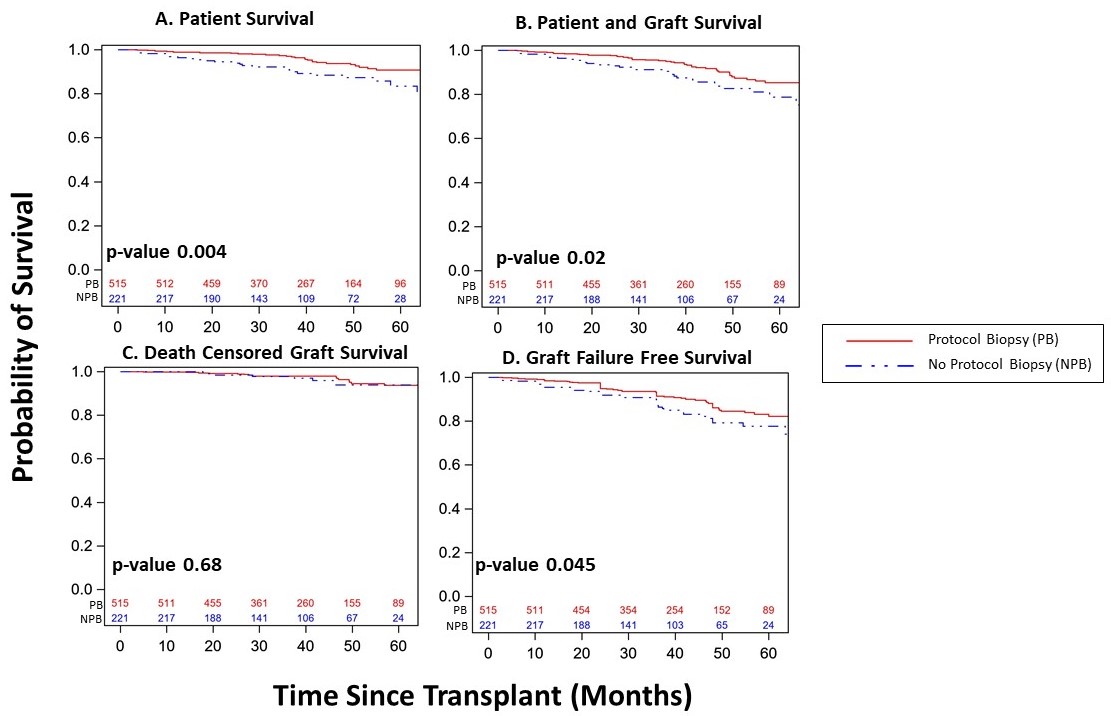


**Figure S3.** Kidney transplant and patient survival for patients with stable clinical course during the 1^st^ year post kidney transplant without protocol biopsy based DSA detection status during the 1^st^ year post-transplant. Kaplan-Meier survival curves demonstrating patient survival (**A,** 83% vs 84%, p=0.92), patient and graft survival (**B,** 79% vs 79%, p=0.93), death censored graft survival (**C,** 95% vs 93%, p=0.76), and Graft Failure free survival (**D**, 76% vs 78%, p=0.82) over study period follow up among patients with stable clinical course during the 1^st^ year post-transplant based on protocol biopsy status.


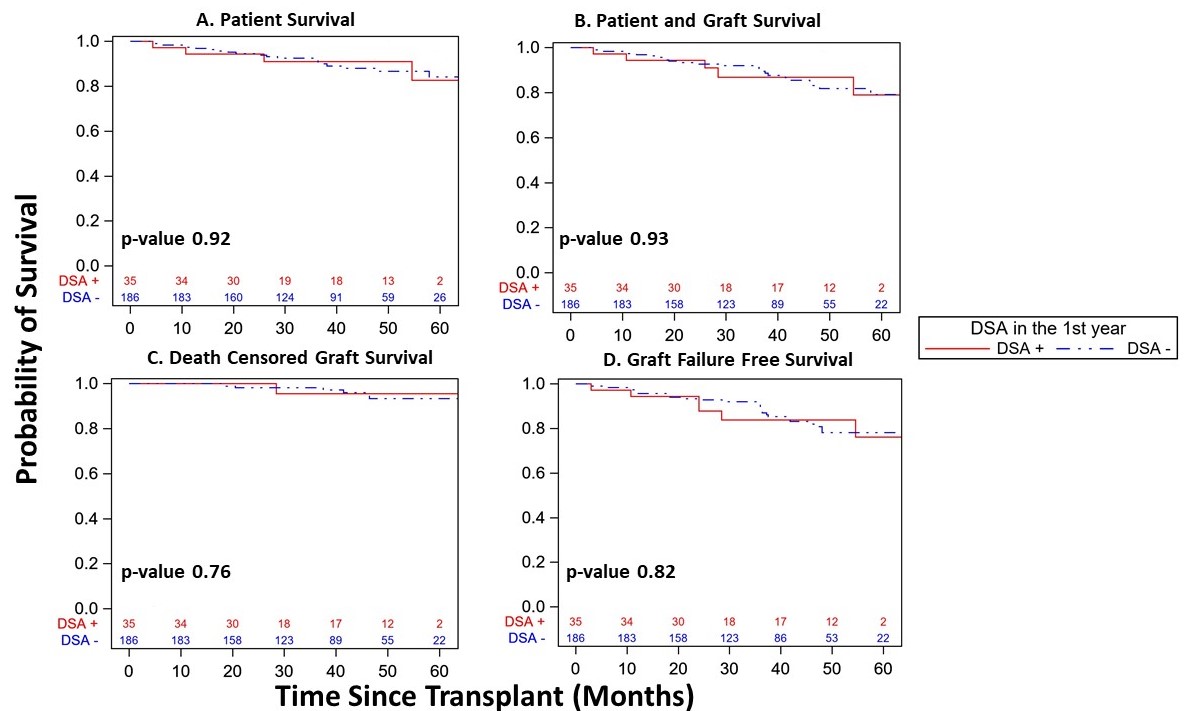


**Figure S4.** Kidney transplant and patient survival for patients with stable clinical course during the 1^st^ year post kidney transplant who had at least one protocol biopsy based DSA detection status during the 1^st^ year post-transplant. Kaplan-Meier survival curves demonstrating patient survival (**A,** 83% vs 92%, p=0.04), patient and graft survival (**B,** 77% vs 87%, p=0.02), death censored graft survival (**C,** 88% vs 95%, p=0.13), and Graft Failure free survival (**D**, 76% vs 84%, p=0.05) over study period follow up among patients with stable clinical course during the 1^st^ year post-transplant based on protocol biopsy status.


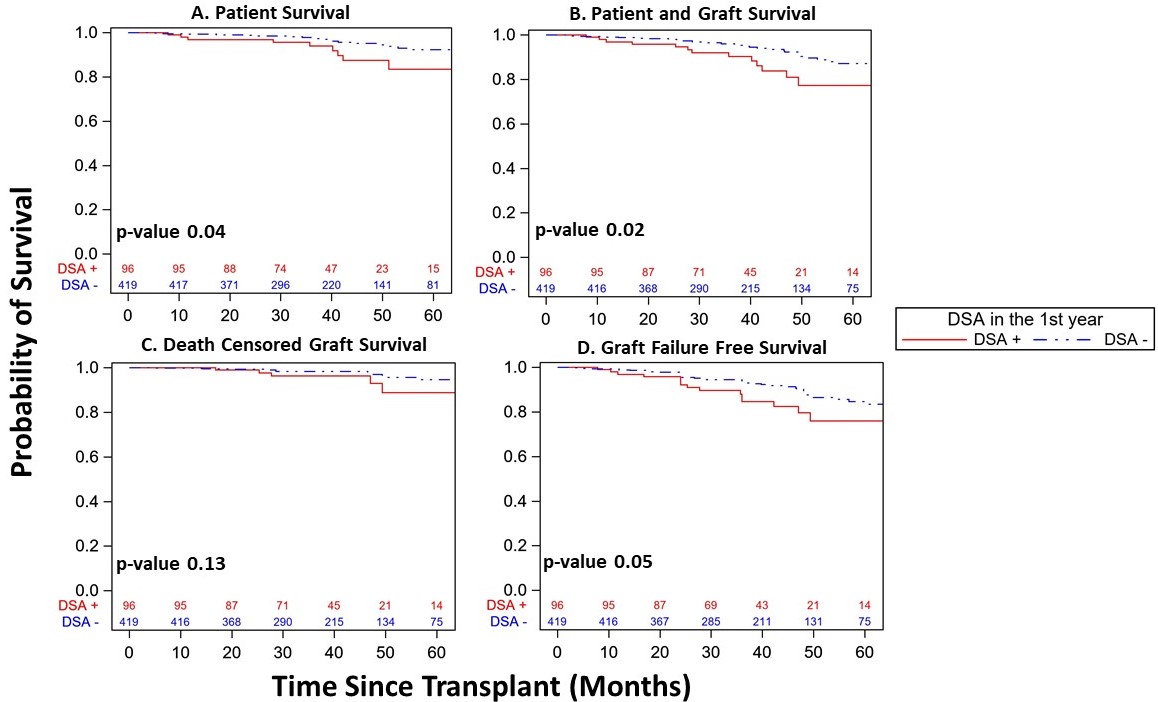


**Figure S5.** Kidney transplant and patient survival for excluded patients with unstable clinical course during the 1^st^ year post kidney transplant. Kaplan-Meier survival curves demonstrating patient survival (**A,** 76% [95% CI 61-86%] vs 86% [95% CI 79-91%], p=0.46), patient and graft survival (**B,** 58% [95% CI 43-71%] vs 74% [95% CI 65-81%], p=0.07), death censored graft survival (**C,** 76% [95% CI 61-86%] vs 86% [95% CI 79-91%], p=0.18), and Graft Failure free survival (**D,** 53% [95% CI 38-66%] vs 61% [95% CI 51-69%], p=0.15) over study period follow up among DSA+ and DSA- patients with unstable clinical course during the 1^st^ year post-transplant.


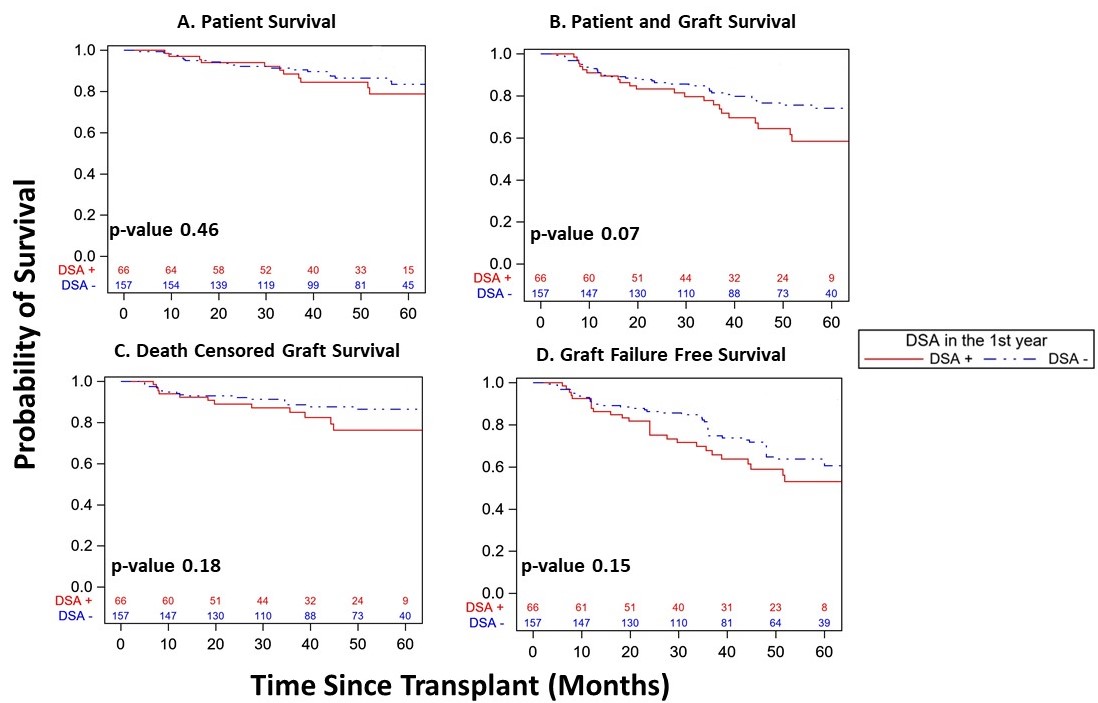

Supplement: Supplementary file 1 [file DataSheet1.docx]
